# Constitutive Expression of Adiponectin in Endothelial Progenitor Cells Protects a Rat Model of Cerebral Ischemia

**DOI:** 10.1155/2017/6809745

**Published:** 2017-10-22

**Authors:** Renwei Zhang, Xiaorui Xie, Qing Yu, Hongliang Feng, Meiyao Wang, Yan Li, Yumin Liu

**Affiliations:** ^1^Department of Neurology, Zhongnan Hospital of Wuhan University, Wuhan 430071, China; ^2^Department of Neurology, Xiangyang Central Hospital, Xiangyang 441000, China

## Abstract

Endothelial progenitor cells (EPCs), as precursors to endothelial cells, play a significant part in the process of endogenous blood vessel repair and maintenance of endothelial integrity. Adiponectin (APN) is an adipocyte-specific adipocytokine. In this study, we aim to test whether we transplant a combined graft of EPCs transfected with the adiponectin gene into a rat model of cerebral ischemia could improve functional recovery after middle cerebral artery occlusion (MCAO). Sprague-Dawley (SD) rats were randomly divided into a MCAO control group, a MCAO EPC treatment group, and a MCAO LV-APN-EPC treatment group. A focal cerebral ischemia and reperfusion model was induced by the intraluminal suture method. After 2 h of reperfusion, EPCs were transplanted by injection through the tail vein. A rotarod test was conducted to assess behavioral function before MCAO and on days 1, 7, and 14 after MCAO. After 14 d, TTC staining, CD31 immunofluorescence, and TUNEL staining were used to evaluate infarct volume, microvessel density, and cell apoptosis. Results revealed that behavioral function, infarct area percentage, microvessel density, and cell apoptosis rates were more favorable in the LV-APN-EPC treatment group than in the EPC treatment group. These data suggested that gene-modified cell therapy may be a useful approach for the treatment of ischemic stroke.

## 1. Introduction

Cerebral ischemic stroke has been recognized as a serious neurological disease that is accompanied by high mortality and morbidity worldwide [1]. To date, most of the neuroprotective compounds developed for ischemic damage are clinically ineffective for acute stroke patients [2, 3]. Although thrombolytic drugs such as tissue-type plasminogen activator (rt-PA) and endovascular interventional therapy have been proven effective, they are also faced with limitations, such as a narrow time window, low dissolution rates, hemorrhage risk, high cost, and other drawbacks. When ischemia occurs, the blood flow in the ischemic zone drops sharply and cannot sufficiently support the metabolic process in brain tissue. The brain is more sensitive than other organs to ischemia and hypoxia. The ischemic zone in cerebral ischemia comprises an ischemic core and a penumbra zone. Cells in the core zone show necrosis, while cells in the penumbra zone show apoptosis. It is important in the treatment of cerebral ischemia to improve the blood flow in the penumbra zone to save the nerve cells.

EPCs, the precursor cells of the vascular endothelium, are able to proliferate and differentiate, participating in neovascularization [4]. This process plays a great role in maintaining the integrity of blood flow to organs and tissues and maintaining endogenous homeostasis. Circulating EPCs can be mobilized endogenously after tissue ischemia and promote neovascularization in the ischemic tissues [5]. Recently, many studies have shown EPC transplantation to have a positive effect in treating cerebral ischemic stroke. However, during the present study, we found that it is difficult to obtain EPCs; meanwhile, under pathological conditions [6–8], the capacity of EPCs for migration, homing, and differentiation was impaired.

Adiponectin is a type of endogenous protein synthesized and secreted by adipose tissue [9]. Research has shown that APN can promote the abundance and functionality of endothelial progenitor cells [10–12]. Therefore, we hypothesized that if we genetically engineered male rat EPCs to overexpress APN in order to maximize the effect of APN on cell number and function, then we could confer better protection against cerebral ischemia.

## 2. Materials and Methods

### 2.1. Preparation of Bone Marrow-Derived EPCs

Ten Sprague-Dawley (SD) male rats weighing 150–180 g, provided by the Centers for Disease Control of Hubei Province, were anesthetized with 10% chloral hydrate and then killed. Fresh bone marrow was harvested aseptically by flushing the tibias and femurs with PBS. Mononuclear cells were isolated by density gradient centrifugation using Ficoll-Paque solution (*d* = 1.077, Sigma, USA), then seeded on T25 plates and cultured in EC basal medium-2 (EBM-2, Lonza, Switzerland) in a humidified atmosphere of 5% CO_2_ at 37°C. Seventy-two hours later, the unattached cells were washed away with media, and the adherent cells were cultured further with changed media.

### 2.2. Characterization of EPCs

We identified EPCs by their property of uptaking acetylated LDL (ac-LDL) and *Ulex europaeus* agglutinin-1 (UEA-1). After 7 days of culturing, we collected EPCs and then put the cells in a confocal culture dish. After 72 h of culturing, we then add 2.4 *μ*g/ml 1,1′-dioctadecyl-3,3,3′,3′-tetramethylindocarbocyanine perchlorate- (Dil-) labeled acetylated LDL (Dil-ac-LDL) and 10 *μ*g/ml fluorescein-isothiocyanate-conjugated *Ulex europaeus* agglutinin- (UEA-) 1 (FITC-UEA-I) to the dish. After culturing the cells in the dark for twelve hours in an incubator, we then observed ac-LDL uptake and lectin binding. Cells with double positive staining were identified as differentiating EPCs.

### 2.3. Gene Transfection

After EPCs were cultured for 7 days and reached 70–80% confluence, the cells were harvested and transfected according to the manufacturer's instructions with lentiviral vectors (Shanghai Genechem Company, China) encoding the human APN gene (LV-APN/EGFP). The lentiviral vector used for this study (Shanghai Genechem Company, China) was Ubi-MCS-3FLAG-SV40-EGFP-IRES-puromycin. To establish the optimal virus concentration for EPC lentiviral transfection, different multiplicities of infection were evaluated according to the lentiviral vector manufacturer's instructions. After our preliminary experiments were performed (data not shown), EPCs were transduced with 100 MOI LV-APN/EGFP for 12 h according to the protocol that we had selected. After genetic transfection, the cells were washed with PBS and cultured with changed media. After 72 h of culture, we visualized enhanced green fluorescent protein (EGFP) by fluorescence microscopy to evaluate the results of the transfection.

### 2.4. APN Expression

To confirm that the APN gene had been successfully transferred, we measured APN expression from EPCs after gene transduction. After 72 h of gene transfection in 6-well plates, we removed the media and washed the cells with PBS. Expression of adiponectin was detected by Western blot analysis. For this procedure, cells were lysed in 200 *μ*l lysis buffer at 1 : 100 ratios and then centrifuged for 10 min at a temperature of 4°C, rotation speed 10,000 r/min. The supernatant was transferred to a new tube. Adiponectin concentration was assessed with a BCA protein assay kit (Biotime, Shanghai, China). Adiponectin was separated by the sodium dodecyl sulfate-polyacrylamide gel electrophoresis (SDS-PAGE) and then transferred to nitrocellulose membranes. The membranes were blocked in 5% nonfat milk in PBS with 0.1% TWEEN 20 or chemiluminescent blocker at room temperature for 1 h. Primary antibodies against adiponectin (1 : 300, Boster, China) and *β*-actin (1 : 500, Boster, China) were used. The membranes were incubated with primary antibody at 4°C overnight. After incubation with 1 : 1000 diluted secondary antibody at 37°C for 1 h, they were incubated in a warm bath with electrochemiluminescence reagent, and 1 min was allowed to elapse before exposure, developing, and fixing. Quantity One software was used for analysis; the integral optical density value was calculated as the quantity of the target protein divided by the quantity of the internal reference protein, *β*-actin. The relative values of protein bands in each group were expressed as the mean ± standard deviation (SD).

### 2.5. Animals and Experimental Groups

Adult male SD rats weighing 280–300 g provided by Hunan Si Lai Ke Jing Da Animal Company were used in all our experiments. All the animals received rodent food and water and were treated humanely. All the rats had free access to water and food. The rats were randomly divided into three groups: the LV-APN-EPC treatment group, the EPC treatment group, and the PBS control group (*N* = 18 per group).

### 2.6. MCAO Model

All rats were anesthetized with 10% chloral hydrate by intraperitoneal injection. Rectal temperature was maintained at 37°C during the surgical procedure using a self-regulating heating pad. The animal experiments were carried out according to the National Institutes of Health Guide for the Care and Use of Laboratory Animals and were approved by the Laboratory Animal Center of Zhongnan Hospital at Wuhan University. The left middle cerebral artery was transiently occluded according to the method of Longa et al. [13, 14] with some modification. Briefly, a midline incision was made on the neck to adequately expose the left common carotid artery (CCA), internal carotid artery (ICA), and external carotid artery (ECA). After ligation of the left CCA and ECA, a 4-0 monofilament nylon thread (Sunbio Biotech, China) was inserted from the CCA into the ICA up to a distance of 18–20 mm. After 2 h of occlusion, the filament was withdrawn for reperfusion.

### 2.7. Cell Transplantation

After gene transfection, the EPCs were cultured for about a week and then harvested in centrifuge tubes. Rats from the LV-APN-EPC treatment group and the EPC treatment group were injected with 2 × 10^6^ cells in 0.5 ml PBS via the tail vein after 2 h of reperfusion of MCAO, while the control group was treated only with 0.5 ml PBS.

### 2.8. Behavioral Test

An accelerating rotarod [15] was used to measure the motor function of the rats. The rats were placed on the rotating rod of the accelerating rotarod apparatus, and the time the animals stayed on the rotarod was recorded. The speed was increased slowly from 5 rev/min to 40 rev/min. A trial ended if the animal fell off the rotating rod or gripped the rotating rod and spun around for two consecutive circuits without attempting to walk on the rungs. All animals were trained three times a day for the 3 days preceding MCAO. The control preischemic data were recorded from three test trials performed immediately before MCAO taken as the internal control. The behavioral test data were presented as percentage of mean time compared with the internal control.

### 2.9. Microvessel Density

Fourteen days after the experiment, animals (*n* = 6, each group) were anesthetized with 10% chloral hydrate, and the brains were fixed by transcardial perfusion with saline. A standard block was obtained from the center of the lesion (bregma −1 mm to +1 mm). Coronal sections (5 *μ*m) were cut with a frozen microtome, and the sections were allowed to air-dry at room temperature for 20 min. They were subsequently treated with 0.1% Triton X-100 (Kerui Biotechnology, China) for 10 min, washed with 0.1 M PBS for 5 min × 3, and then blocked with 1% bovine serum albumin (BSA) for 15 min. The sections were incubated with the primary antibody (CD31, Abcam, 1 : 200) overnight. After being washed, the sections were incubated with Cy3-conjugated secondary antibodies (1 : 10,000, Aspen, China) for 1 h. After being washed with PBS, the sections were treated with DAPI (Sigma, America). We counted CD31-positive cells per field to evaluate microvessel density (MVD) in the ischemic boundary zone (IBZ) by fluorescence microscopy.

### 2.10. TUNEL Staining

Fourteen days after the experiment, 6 rats were randomly selected from each group and sacrificed with 10% chloral hydrate. The brains were fixed by transcardial perfusion with saline, followed by perfusion with an immersion in 4% paraformaldehyde overnight at 4°C. A standard paraffin block was obtained from the center of the lesion (bregma −1 mm to +1 mm). A series of 8 *μ*m thick sections were cut from the block with a microtome. Five sections were randomly chosen for terminal deoxynucleotidyl transferase-mediated dUTP-biotin nick end labeling (TUNEL) staining to identify apoptotic cells in the IBZ. We randomly selected 5 microscopic fields in the ischemic border zone and counted the TUNEL-positive cells using a TUNEL kit (Roche, Switzerland). To represent the results of TUNEL staining, we calculated the apoptosis rate using the following formula: apoptosis rate = positive cells/total cells per field × 100%.

### 2.11. Infarct Volume Assessment

Rats were decapitated on day 14 (each group, *n* = 6) after MCAO, and the brains were removed after transcardial perfusion fixation with saline. A series of 2 mm thick coronal sections were obtained using a brain matrix. The slices were immediately immersed in 2% 2,3,5-triphenyl tetrazolium chloride (TTC) (Sigma, America) at 37°C for 20 min and then treated with 4% paraformaldehyde. The white area in each section was defined as the infarct zone, and the infarct volume was evaluated by ImageJ software (NIH). To represent infarct volume, we calculated the infarct rate using the following formula: infarct rate = (area of contralateral hemisphere − area of normal region in the ipsilateral hemisphere)/area of contralateral hemisphere × 100% [16].

### 2.12. Statistical Analysis

The results were presented as the mean ± standard error of mean. The statistical analysis was performed using SPSS 19.0. One-way analysis of variance followed by a Student-Newman-Keuls *q*-test was applied to analyze the statistical significance of differences between groups. *P* < 0.05 is considered statistically significant.

## 3. Results

### 3.1. EPC Morphology

**Figure 1:**
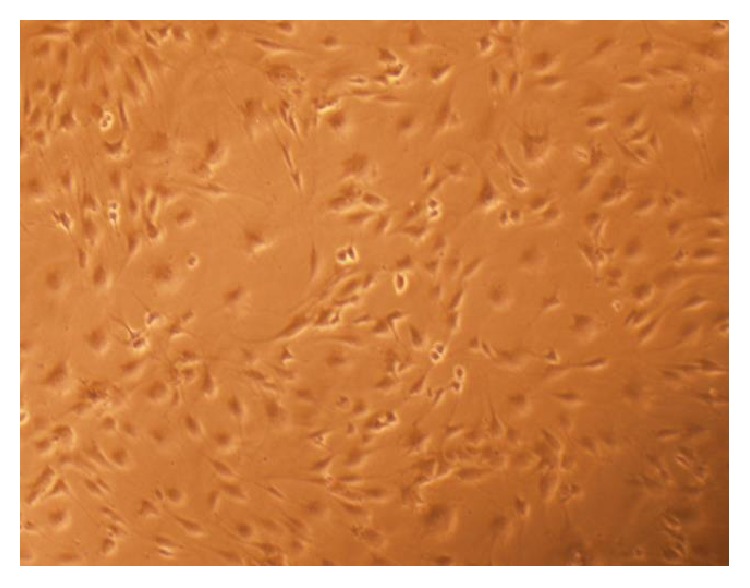
Two weeks after culture, the cells displayed a cobblestone-like morphology (×100).

After 2 weeks of culture, the cells displayed a cobblestone-like shape (Figure 1).

### 3.2. EPC Characterization

**Figure 2:**
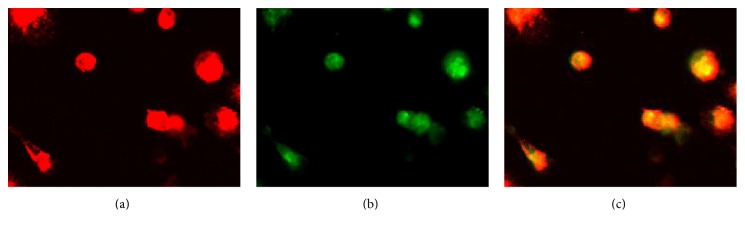
Uptake of Dil-acLDL shown in red (a); FITC-UEA-1 binding shown in green (b); double Dil-acLDL/FITC-UEA-1 positive shown in yellow (c).

EPCs, as precursors to endothelial cells, have the typical endothelial cell functions of uptaking ac-LDL intake and UEA-1. After 7 days of culture, we observed that the cells were double positive for Dil-ac-LDL uptake and FITC1-UEA-1 binding (Figure 2).

### 3.3. Transfection Effect

**Figure 3:**
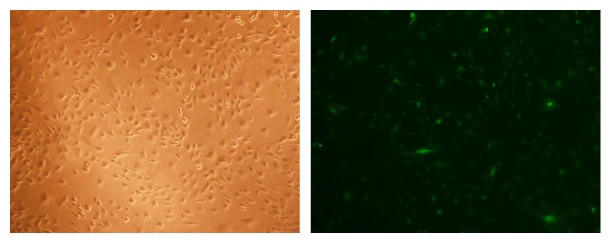
Three days after gene transfection, we could observe the EGFP expression in a fluorescence microscope (×100).

Three days after gene transfection, EGFP expression was visible by fluorescence microscopy (Figure 3).

### 3.4. APN Expression

**Figure 4:**
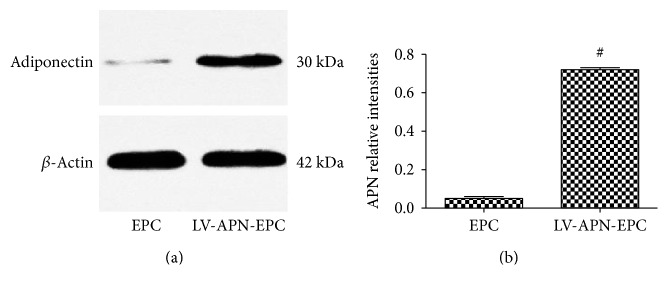
The expression of APN from APN gene-modified EPCs and unmodified EPCs by Western blot analysis after 72 h of gene transfection (a). APN gene-modified EPCs expressed high levels of APN, while unmodified EPCs expressed very little APN. Compared with unmodified EPCs, the APN expression was elevated in the gene-modified cells (*P* < 0.05). Experimental data are presented as mean ± standard error of mean. Differences between groups are assessed by one-way analysis of variance. Versus EPC treatment group, ^#^*P* < 0.05 (b).

After 72 h of gene transfection, we used Western blot analysis to test the gene expression of APN. We found that APN gene-modified EPCs expressed high levels of APN, while unmodified EPCs expressed very little APN. Compared with unmodified EPCs, the APN expression was elevated in the gene-modified cells (*P* < 0.05) (Figure 4).

### 3.5. Behavioral Function

**Figure 5:**
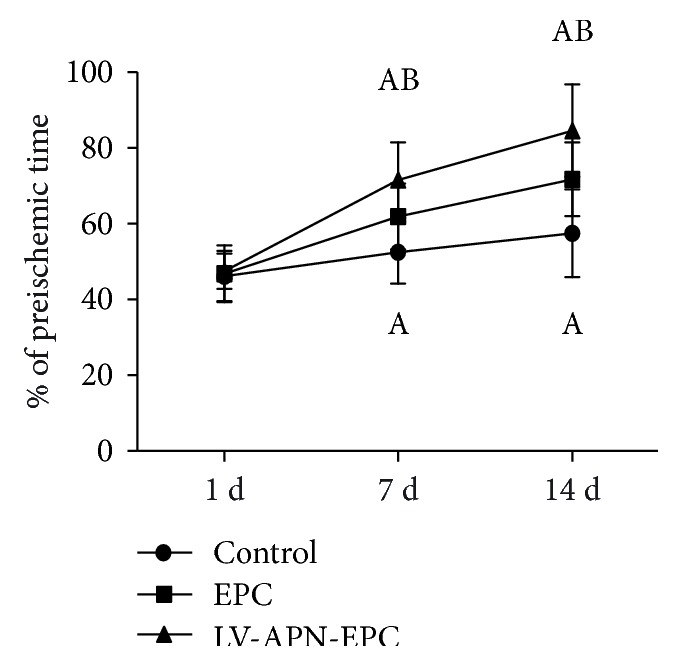
On day 1, no significance was found among three groups. On days 7 and 14, the behavioral function of LV-APN-EPC and EPC treatment groups was improved compared with the PBS group (*P* < 0.05), while the behavioral function of the LV-APN-EPC treatment group was improved compared with the EPC treatment group (*P* < 0.05). Experimental data are presented as mean ± standard error of mean. Differences between groups are assessed by one-way analysis of variance followed by Student-Newman-Keuls *q*-test (*N* = 8 per group). Versus control group, ^a^*P* < 0.05; versus EPC treatment group, ^b^*P* < 0.05.

On day 1, no significance was found among the three groups. On days 7 and 14, the behavioral function of the LV-APN-EPC and EPC treatment groups was improved compared with the PBS group (*P* < 0.05), while the LV-APN-EPC treatment group was improved compared with the EPC treatment group (*P* < 0.05) (Figure 5()).

### 3.6. Microvessel Density

**Figure 6:**
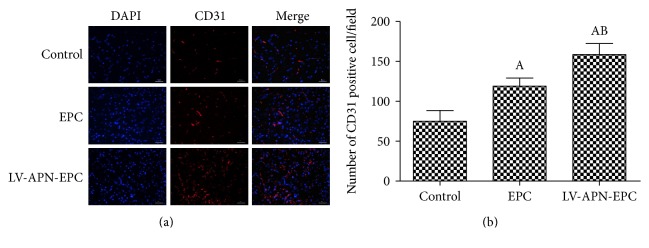
CD31 staining positive cells were used to represent microvessel density in the IBZ (a). Microvessel density in the IBZ from LV-APN-EPC and EPC treatment groups was elevated compared with the PBS group (*P* < 0.05), while that of the LV-APN-EPC treatment group was elevated compared with the EPC treatment group (*P* < 0.05). Experimental data are presented as mean ± standard error of mean. Differences between groups are assessed by one-way analysis of variance followed by Student-Newman-Keuls *q*-test (*N* = 6 per group). Versus control group, ^a^*P* < 0.05; versus EPC treatment group, ^b^*P* < 0.05 (b).

Microvessel density in the IBZ was elevated in the LV-APN-EPC and EPC treatment groups compared with the PBS group (*P* < 0.05), while that of the LV-APN-EPC treatment group was elevated compared with the EPC treatment group (*P* < 0.05) (Figure 6).

### 3.7. Cell Apoptosis Rate

**Figure 7:**
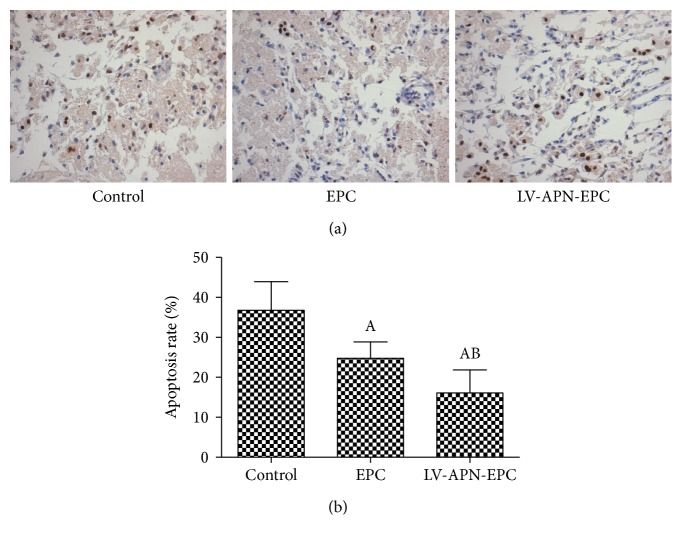
TUNEL staining was used to detect the cell apoptosis of every group (a). Cell apoptosis rate from LV-APN-EPC and EPC treatment groups was decreased compared with the control group (*P* < 0.05), while that of the LV-APN-EPC treatment group was decreased compared with the EPC treatment group (*P* < 0.05). Experimental data are presented as mean ± standard error of mean. Differences between groups are assessed by one-way analysis of variance followed by Student-Newman-Keuls *q*-test (*N* = 6 per group). Versus control group, ^a^*P* < 0.05; versus EPC treatment group, ^b^*P* < 0.05 (b).

TUNEL staining showed that cell apoptosis rate in the LV-APN-EPC and EPC treatment groups was decreased compared with the control group (*P* < 0.05), while that of the LV-APN-EPC treatment group was decreased compared with the EPC treatment group (*P* < 0.05) (Figure 7).

### 3.8. Infarction Percentage

**Figure 8:**
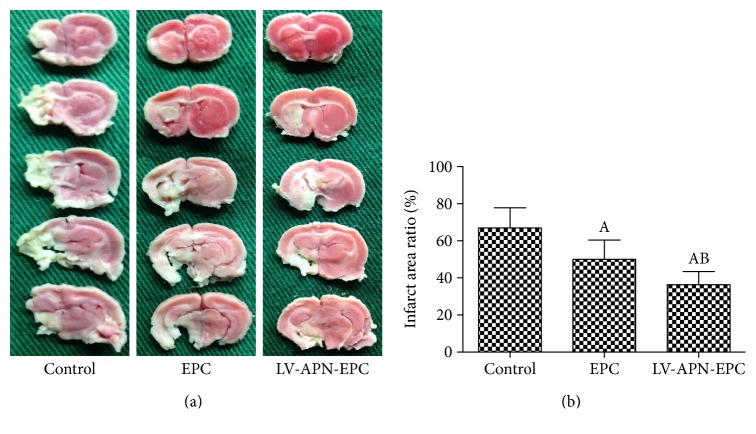
14 d after the experiment, TTC staining showed the infarction of every group (a). The infarction percentage of LV-APN-EPC and EPC treatment groups decreased compared with the PBS group (*P* < 0.05), while the LV-APN-EPC treatment group had a lower infarction percentage than the EPC treatment group (*P* < 0.05). Experimental data are presented as mean ± standard error of mean. Differences between groups are assessed by one-way analysis of variance followed by Student-Newman-Keuls *q*-test (*N* = 6 per group). Versus control group, ^a^*P* < 0.05; versus EPC treatment group, ^b^*P* < 0.05 (b).

TTC staining showed that the infarction percentage from the LV-APN-EPC and EPC treatment groups was decreased compared with that of the PBS group (*P* < 0.05), while the LV-APN-EPC treatment group had a lower infarction percentage than the EPC treatment group (*P* < 0.05) (Figure 8).

## 4. Discussion

In our experiment, we generated a middle cerebral artery occlusion and reperfusion model using the protocol described by Longa et al. [13]. After the LV-APN-EPCs were administered to the rats through the tail vein, we found that behavioral function was improved, infarct volume rates were decreased, IBZ microvascular density was increased, and cell apoptosis rates were decreased and were more favorable than the corresponding results from the EPC treatment group. From the results of CD31 immunofluorescence staining, we found that IBZ microvascular density was increased, which indicated that microvessels play a crucial mechanistic role in protecting against cerebral ischemic disease.

Revascularization is an important process to restore impaired blood vessels in the body. Two different forms of revascularization can occur in the body, namely, angiogenesis and vasculogenesis. Angiogenesis means new vessels are produced from mature endothelial cells of preexisting vessels [17], while vasculogenesis refers to the growth of new blood vessels from endothelial cells acting as precursor cells, such as angioblasts or EPCs [18]. EPCs derived from bone marrow can circulate in the peripheral blood and are known to play a significant part in the process of endogenous blood vessel repair and maintenance of endothelial integrity.

Recently, EPC transplantation has shown a significant positive effect in the treatment of many kinds of ischemic disease, including ischemic cerebral infarction, with the features of localizing to the ischemic area, proliferating and participating in vasculogenesis. Zhang et al. [19] treated a MCAO model with bone marrow-derived EPCs and confirmed that EPCs could form new blood vessels through vasculogenesis. Fan et al. [20] found that EPCs could improve behavioral function and decrease infarct area through an experiment in which EPCs were transplanted into a MCAO model. Kawamoto et al. [21] obtained EPCs from human peripheral blood, expanded them ex vivo, and then injected them intravenously into a rat model of myocardial ischemia. They found that EPCs could significantly decrease myocardial infarct area, increase capillary density in the myocardial infarct zone, and play an important role in the preservation of left ventricular function.

From our experiment, we confirmed that both EPC treatment and LV-APN-EPCs treatment could increase microvessel density and improve neurological function, thereby protecting against the effects of ischemic stroke. The likely mechanism is that hypoxic ischemic brain tissue released inflammatory mediators, which caused EPCs to home to the hypoxic ischemic brain tissue, and then the EPCs proliferated and differentiated into endothelial cells, which participated in vascular repair and neovascularization and helped restore blood flow and neurological function.

However, in the course of the study, we also found that it is difficult to obtain EPCs; meanwhile, under pathological conditions [7, 22], the capacity of EPCs for migration, homing, and differentiation was impaired. Maintenance of normal numbers and function of EPCs in the body circulation is now regarded as an important novel endogenous vascular repair factor [23, 24]. Genetic modification is a prospective method to enhance the capacity of EPCs for migration, proliferation, and differentiation.

Adiponectin is an adipocyte-specific adipocytokine with antiatherosclerotic [25] and anti-inflammatory features [26]. In the current study, we found that adiponectin could increase the abundance and improve the function of EPCs. Nakamura et al. [27] found that adiponectin can promote the migration activity of EPCs, mainly through PI 3-kinase/Cdc42/Rac1. Huang et al. [28] found that globular adiponectin could ameliorate high glucose-impaired EPC function in vasculogenesis by restoring eNOS activity and improved high glucose-impaired EPC function by NO- and p38 MAPK-related mechanisms. Eren et al. [11] found that adiponectin could improve the resistance of EPCs to apoptosis, and this effect may be mediated by XIAP (X-linked inhibitor of apoptosis protein). Chang et al. [12] found that adiponectin prevents senescence and apoptosis of endothelial progenitor cells by suppressing the p38 MAP kinase/p16INK4a signaling pathway. Ouchi et al. [10] found that adiponectin stimulated the differentiation of human umbilical vein endothelium cells (HUVECs) into capillary-like structures in vitro and functioned as a chemoattractant in migration assays. Adiponectin promoted the phosphorylation of AMP-activated protein kinase (AMPK), Akt/protein kinase B, and endothelial nitric oxide synthase (eNOS) in HUVECs. Thereby, in this study, we genetically engineered EPCs with APN gene, and our data showed that gene-modified EPCs could overexpress adiponectin.

In the present study conducted with isolated and cultivated EPCs and MCAO rats, we observed the effect of APN gene-modified EPCs on a rat model of ischemic stroke. EPCs transfected with the APN gene resulted in improved function and reduced ischemic damage in the rat model of MCAO compared with the EPC treatment group and the control group. These data suggest that gene-modified cell therapy may be a useful method for the treatment of ischemic stroke. The likely mechanism was that APN improved the proliferation and differentiation function of EPCs through signaling pathways involved in vascular endothelial repair and that it participated in neovascularization, increased the microvessel density, and improved tissue blood flow. In addition to the present work, many other studies about gene modification of EPCs have suggested that transplantation of gene-modified EPCs is a highly effective method [29, 30].

In conclusion, our experiment revealed that behavioral function, infarct area percentage, microvessel density, and cell apoptosis rates were more favorable in the LV-APN-EPC treatment group than in the EPC treatment group. These data suggested that gene-modified cell therapy may be a useful approach for the treatment of ischemic stroke.
